# Diurnal Variation of 8-hydroxy-2’-deoxyguanosine in Continuous Time Series of Two Breast Cancer Survivors

**DOI:** 10.5334/jcr.252

**Published:** 2025-05-19

**Authors:** Joschua Geuter, Lennart Seizer, Germaine Cornelissen Guillaume, Ayse Basak Engin, Dietmar Fuchs, Christian Schubert

**Affiliations:** 1 Sainsbury Wellcome Centre, London, United Kingdom; 2 Department of Child and Adolescent Psychiatry, Psychosomatics and Psychotherapy, University Hospital of Tübingen, Tübingen, Germany; 3 Halberg Chronobiology Center, University of Minnesota Medical School, Minneapolis, Minnesota, United States of America; 4 Department of Toxicology, Faculty of Pharmacy, Gazi University, Ankara, Turkey; 5 Institute of Biological Chemistry, Biocenter, Medical University Innsbruck, Innsbruck, Austria; 6 Department of Psychiatry, Psychotherapy, Psychosomatics and Medical Psychology, Medical University Innsbruck, Innsbruck, Austria

**Keywords:** 8-OHdG, breast cancer, circadian rhythm, time series analysis, integrative single-case study, oxidative stress

## Abstract

8-hydroxy-2’deoxyguanosine (8-OHdG) is an oxidative product removed from DNA following radical oxygen species-induced damage. As a water-soluble molecule, it can be measured non-invasively in urine and is commonly used as a marker for ‘whole-body’ oxidative stress. However, its validity and reliability in assessing oxidative stress across various chronic diseases and in early carcinogenesis screening in clinical diagnosis and research remain subjects of debate. To determine optimal measurement timing and duration, it is essential to establish the circadian profile of 8-OHdG under everyday life conditions. Here, applying the integrative single-case design, we show the presence of day-night differences for 8-OHdG in continuous time series of two breast cancer survivors while considering different correction approaches. The participants sampled their urine in 12-h-pooled collections over one month. In both subjects, 8-OHdG levels were significantly higher during the day than at night regardless of whether they were corrected by creatinine or urine volume (creatinine corrected: t = –6.43, *p* < 0.01 [subject 1], t = –2.69, *p* = 0.01 [subject 2]; volume corrected: t = –7.30, *p* < 0.01 [subject 1], t = –3.69, *p* < 0.01 [subject 2]). Notably, urinary 8-OHdG levels show higher variability in night samples compared to day samples. These findings underscore the importance of considering daily fluctuations in 8-OHdG levels in both clinical and research studies, as well as the need to account for the dynamic characteristics of stress markers to minimize the risk of inconsistent or misleading results in clinical diagnostics.

## Introduction

Radical oxygen species (ROS) are highly reactive molecules naturally generated during cellular metabolism. However, their levels can be further increased by environmental factors such as (psychological) stress, tobacco smoke, or radiation [1,2,3,4,5,6,7], leading to cellular damage and contributing to aging and disease. If not neutralized, ROS can attack lipids, proteins, and DNA [8,9,10,11,12]. Under normal physiological conditions, there is a balance between endogenous oxidants and antioxidants. However, oxidative stress occurs when ROS production exceeds the body’s antioxidant capacity [1,10].

8-hydroxy-2’-deoxyguanosine (8-OHdG) is a prominent marker of ROS-induced oxidative DNA damage that is formed by the oxidation of guanosine [5,13,14,15]. After oxidation, repair mechanisms remove 8-OHdG from the DNA [9,18]. As a free deoxynucleoside, it is water-soluble and excreted into urine, reflecting the balance between oxidative damage and DNA repair [16,17]. Compared to the direct monitoring of ROS levels, which, although playing a significant role in tumorigenesis, are less stable and thus challenging to accurately measure, these more stable metabolites resulting from ROS damage are the preferred biomarkers [18]. Furthermore, the non-invasive measurement of 8-OHdG in urine offers a significant advantage over other biomarkers, contributing to its extensive study in various health conditions, including cancer, neurodegenerative disorders, and chronic diseases [19,20,21,22,23]. In breast cancer patients, 8-OHdG levels are significantly higher in cancerous tissues but tend to decrease in more advanced clinical stages [16,24]. Additionally, increased levels of 8-OHdG in breast cancer tumor tissues are associated with poor overall survival [18]. 8-OHdG thus is a promising biomarker in predicting the disease prognosis and survival rate for breast cancer patients [18,25].

Despite its potential use as a biomarker for breast cancer, 8-OHdG’s excretion dynamics remain poorly understood, with conflicting reports about its circadian rhythm [18,29,30,31,32,33,34]. To address this question, and further understand the possibly altered oxidative stress profile in breast cancer survivors after tumor treatment, we analyzed urinary 8-OHdG level dynamics corrected by either creatinine concentration or urine volume in two breast cancer survivors participating in integrative single-case studies [26,27]. This study design aims to capture the natural ebb and flow of everyday life to investigate the dynamic behavior of the variables under conditions of “life as it is lived” [28,29,30,31]. Cross-sectional studies that often rely on sampling within a single day fail to capture intra-individual variability, which can be achieved by intensive longitudinal designs that offer a novel approach to overcome these limitations, allowing robust detection of diurnal variations across days. By collecting 12-h pooled urine samples over extended periods (32 and 28 days) from two breast cancer survivors, we aim to detect day-night differences in urinary 8-OHdG levels regardless of whether creatinine or volume correction was applied. This insight could guide the future use of 8-OHdG as a biomarker for breast cancer and its use to assess the long-term treatment effects on oxidative stress in breast cancer survivors.

## Material and Methods

### Study Design

This study analyzed the time series data from two previous integrative single case studies involving breast cancer survivors (see [26,27]). For subject 1, the study extended over 32 days or 63 12-h intervals (from December 7th, 2004, to January 7th, 2005), while for subject 2, it spanned 28 days or 55 12-h intervals (from July 13th to August 9th, 2006). To ensure high ecological validity, minimal interference was made in the subjects’ regular daily routine. The subjects each collected their entire urine, dividing it into two periods: day (from ~08:00 to ~20:00) and night (~20.00 to ~08:00). Upon collection, 0.5 g sodium metabisulfite and 0.5 g disodium dihydrogen ethylenediaminetetraacetate (Sigma-Aldrich, United States) were added to the polyethylene collection canisters preventing urine sedimentation and oxidation. At the end of each period, the subjects recorded the total urine volume per 12-h period, and several aliquots were directly frozen at –20°C in 2 ml Eppendorf tubes. Once a week, samples were brought to the laboratory and stored at –70°C until further analysis. For a more detailed study design description, see [28,31].

The 12-h pooled sampling intervals were selected to optimize the biological representation while maintaining high ecological validity. By capturing the entirety of the daily urine output, we obtain a comprehensive metabolic profile, while minimizing the interference with normal daily activities and allowing the “life as it is lived” approach. Compared to shorter sampling intervals, 12-h pooled sampling minimizes the participant’s burden while ensuring samples that capture diurnal variations.

Both subjects provided written informed consent to participate in the study and for data publication. The Ethics Review Committee of Hannover and Freiburg University approved the design.

### Inclusion and Exclusion Criteria

The inclusion criteria for this study comprised individuals diagnosed with early resectable breast cancer at stages 0, I, or II, who have successfully completed local and/or systemic adjuvant cancer therapy. Subjects should have received their initial breast cancer diagnosis between 3 and 7 years before enrollment and must be clinically free from evidence of metastatic or recurrent lesions. Additionally, eligible participants must be 18 years or older, postmenopausal, able to read and write German, and capable of providing informed consent study participation and data publication.

Exclusion criteria for this study involved individuals with a history of immunologically or endocrinologically related diseases that may impact the endocrine or immune system, as well as a history of psychiatric hospitalization or severe psychological distress within the last 6 months. Additionally, individuals regularly using medications that could influence the biochemical or psychological parameters under investigation (such as immunosuppressants, tamoxifen, or antidepressants) or consuming excessive amounts of alcohol (defined as more than 15 alcoholic beverages per week) are excluded.

### Subject Description

Subject 1, a 60-year-old woman, was married with no children. She was a non-smoker and consumed moderate amounts of alcoholic beverages. Five years before the study, she was diagnosed with ductal mammary carcinoma in her left breast (pT1c, N0, M0, G2, Her2-/neu receptor-positive). The therapy included two months of radiotherapy, followed by breast-conserving surgery. After therapy, for 1.5 years, she experienced cancer-related fatigue and symptoms of depression. One year before the study, her left breast was removed after a recurrence diagnosis. Adjuvant therapy consisted of tamoxifen (anti-estrogen) up to the recurrence diagnosis followed by anastrozole (Arimidex), an aromatase inhibitor.

Subject 2, a 49-year-old woman, was married and had three children. She was a non-smoker, consumed moderate amounts of alcoholic beverages, and suffered from chronic depression (dysthymia, F34.1). Five years before the study, she was diagnosed with ductal breast cancer (C50.4) in the right breast (pT2, pN1biv (6 of 13), cM0, G3, R0, ER 10%, PR 70–80%, HER2+/neu+, score = 3). Her right breast was surgically removed in 2001 (mastectomy and lymphadenectomy). Additional therapy included radiotherapy, chemotherapy according to the EC scheme (100 mg Epirubicin and 1000 mg Cyclophosphamide), and tamoxifen treatment (completed six months before the study began). After chemotherapy, she was diagnosed with secondary amenorrhea. Shortly after cancer diagnosis and therapy, the patient developed severe cancer-related fatigue and a substantial increase in depression intensity with clear functional impairment (adjustment disorder with depressed mood, F43.21), both lasting until study start. The only medication the patient took was aspirin on an irregular basis.

At the study’s outset, careful medical examinations confirmed that both subjects were clinically free of metastatic or recurrent lesions.

### Biochemical Measurements

Urinary 8-OHdG concentrations were analyzed using enzyme-linked immunosorbent assays (ELISA; Kit Cayman, USA). According to the manufacturer, cross-reactivity occurs with 8-hydroxyguanosine (23%), 8-hydroxyguanine (23%), and guanosine (<0.01%). Urinary creatinine levels were determined via high-pressure liquid chromatography (Model LC 550; Varian Associates, Palo Alto, CA) [32]. Each sample was measured at least twice using different aliquots and the resulting measurements were subsequently averaged. All urinary aliquots from each subject were measured in a single run.

8-OHdG levels are expressed in ng per mg urinary creatinine to correct for urinary flow and glomerular filtration rate (creatinine correction) or µg per h excreted on average per pooled 12-h urine (volume correction) to correct for hydration status [33].

### Statistical Analysis

All statistical analyses were conducted in *R* 4.2 [34], Excel 2016, or in-house programs [31]. Pearson correlations were calculated to quantify the linear relationship between creatinine- and volume-corrected levels of urinary 8-OHdG, allowing assessment of whether both correction approaches yield comparable results, assuming linear relationship between these variables. Paired t-tests were computed to compare urinary 8-OHdG levels during day and night periods within the same subjects, assuming normal distribution between paired observations. This approach can reveal diurnal differences, although not more complex temporal patterns. To characterize the temporal relationship between successive measurements and provide insights into potential rhythmic patterns, autocorrelation functions (ACF) of the time series were computed. This allows measuring the correlation of the 8-OHdG time series with delayed copies of itself [35], assuming stationarity of the time series. For periodic pattern analysis, one-way analysis of variance (ANOVA) was performed on samples grouped in consecutive 4-day intervals (8 timepoints per interval), followed by least squares spectral analysis [36] using 30 days as a fundamental period (average recording length) to specifically test for the presence and characteristics of periodic components. In all analyses, statistical significance was considered at *p* < 0.05. The study duration was calculated to include at least 50 equidistant measurements per subject according to Box *et al*. 2015, ensuring sufficient statistical power to detect physiologically meaningful changes in urinary 8-OHdG levels.

### Data Availability

The data generated in this study are available in the supplementary material. The code is available at GitHub https://github.com/Joschua21/Diurnal-variations-of-8-hydroxy-2-deoxyguanosine-in-two-breast-cancer-survivors.

## Results

### Day-Night Variability in 12-h pooled 8-OHdG

**Figure 1:**
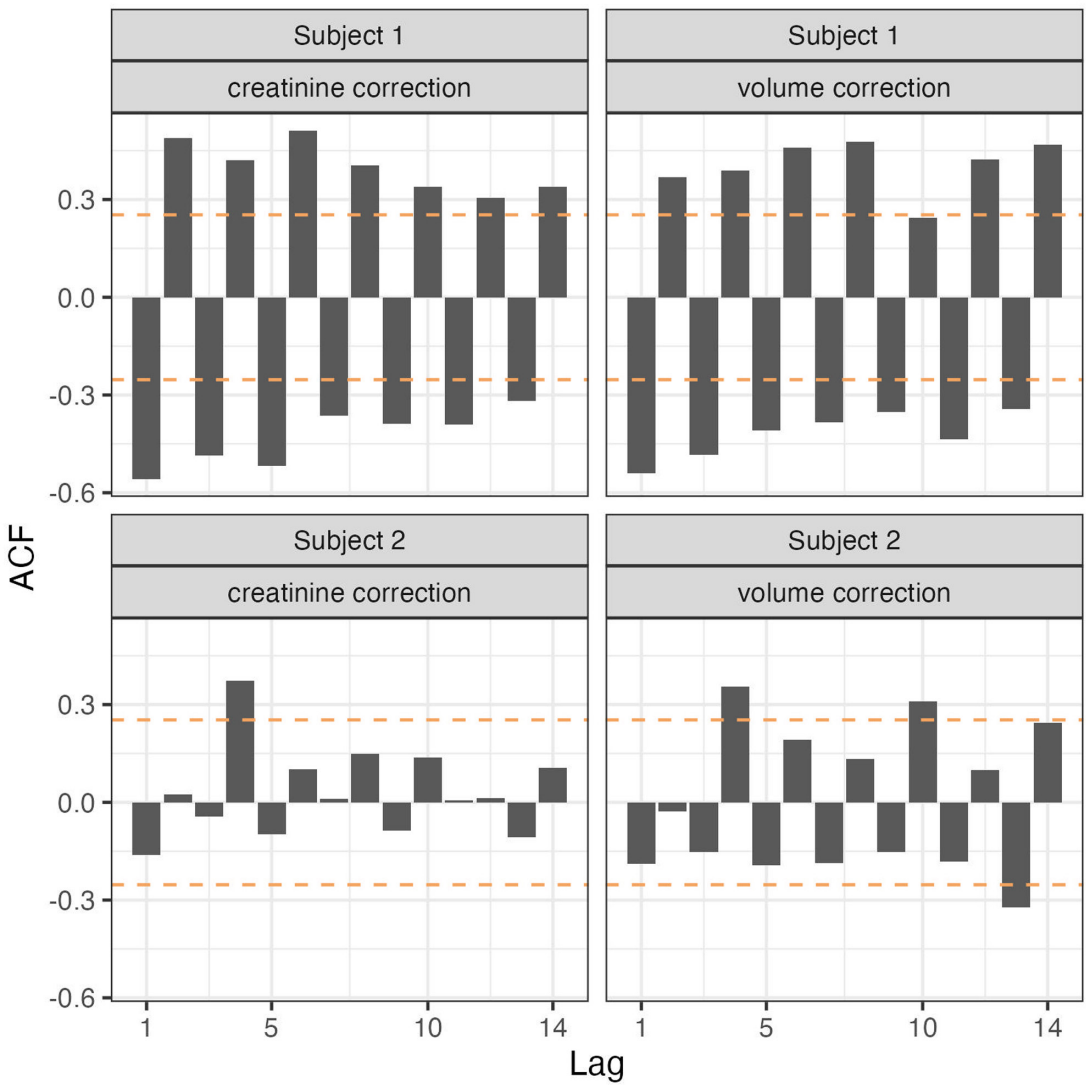
**Autocorrelation function (ACF) of urinary 8-OHdG**. Up to 14-lags for subject 1 (top row) and subject 2 (bottom row) are shown, using either creatinine- (left column) or urinary volume-correction (right column). Coefficients (bars) reaching the upper or lower confidence limits (dotted lines) are significant at *p* < 0.05.

**Figure 2:**
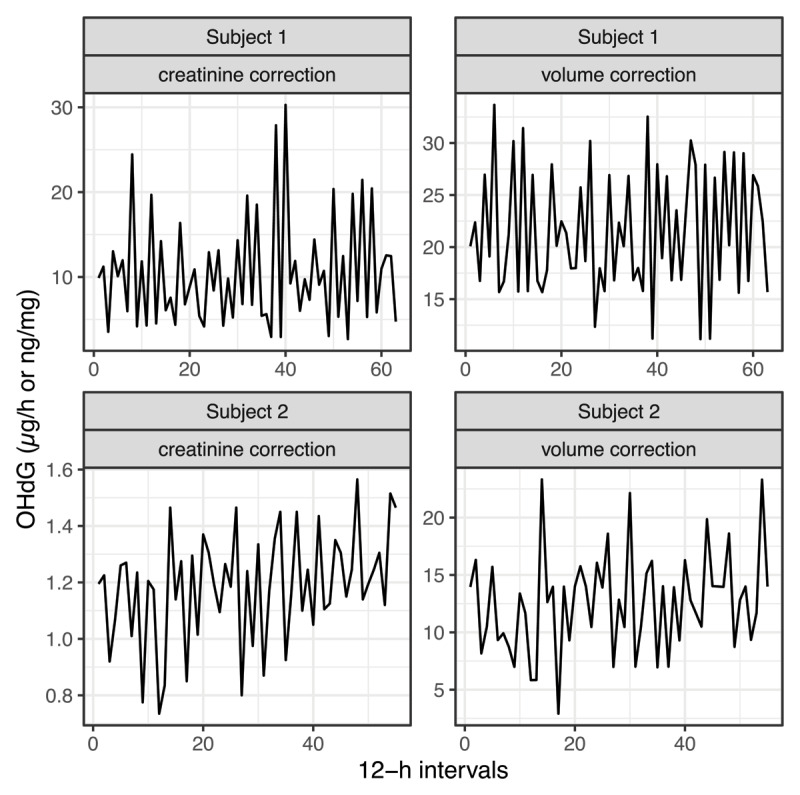
**Time series of urinary 8-OHdG**. The time series of urinary 8-OHdG are given as creatinine-corrected (ng/mg creatinine; left column) and volume-corrected (µg/h; right column) values for subject 1 (n = 63 12-h intervals; top row) and subject 2 (n = 55 12-h intervals; bottom row).

The levels of creatinine- and volume-corrected 8-OHdG in consecutive 12-h pooled urine collections for both subjects are depicted in Figures 1 and 2, while the corresponding descriptive statistics for these time series are shown in Table 1. Notably, there were no missing data. Correlations between values from the two correction approaches, volume- and creatinine corrected 8-OHdG levels, were significantly positive in both subjects (both: *r* = 0.73, *p* < 0.01). In both subjects and with both correction approaches, urinary 8-OHdG levels exhibited higher variability in night samples compared to day samples (12 h % CV, Table 1).

**Table 1:** **Descriptive statistics of urinary 8-OHdG**. Descriptive statistics (mean, standard deviation, range) of creatinine- (ng 8-OHdG per mg creatinine) and volume-corrected (µg 8-OHdG excreted per hour) values of urinary 8-OHdG for both subjects (S1 = Subject 1, S2 = Subject 2) are shown for the entire study duration. Coefficients of variation in percentage points (% CV) are given for 12 h, 24 h, and 48 h collection periods.


	CORRECTION	UNIT	MEAN	RANGE	% CV			

					12 h (DAY)	12 h (NIGHT)	24 h	48 h

***S1***	Creatinine	ng/mg	10.43 ± 6.36	11.16 – 33.67	40.68	40.96	28.58	19.75

	Volume	µg/h	21.51 ± 5.88	2.68 – 30.29	18.32	22.10	13.11	7.90

***S2***	Creatinine	ng/mg	1.19 ± 0.20	0.74 – 1.57	13.63	17.89	10.77	7.12

	Volume	µg/h	12.61 ± 4.34	2.90 – 23.33	30.04	31.78	22.36	13.48


### Diurnal Variations of 8-OHdG

**Figure 3:**
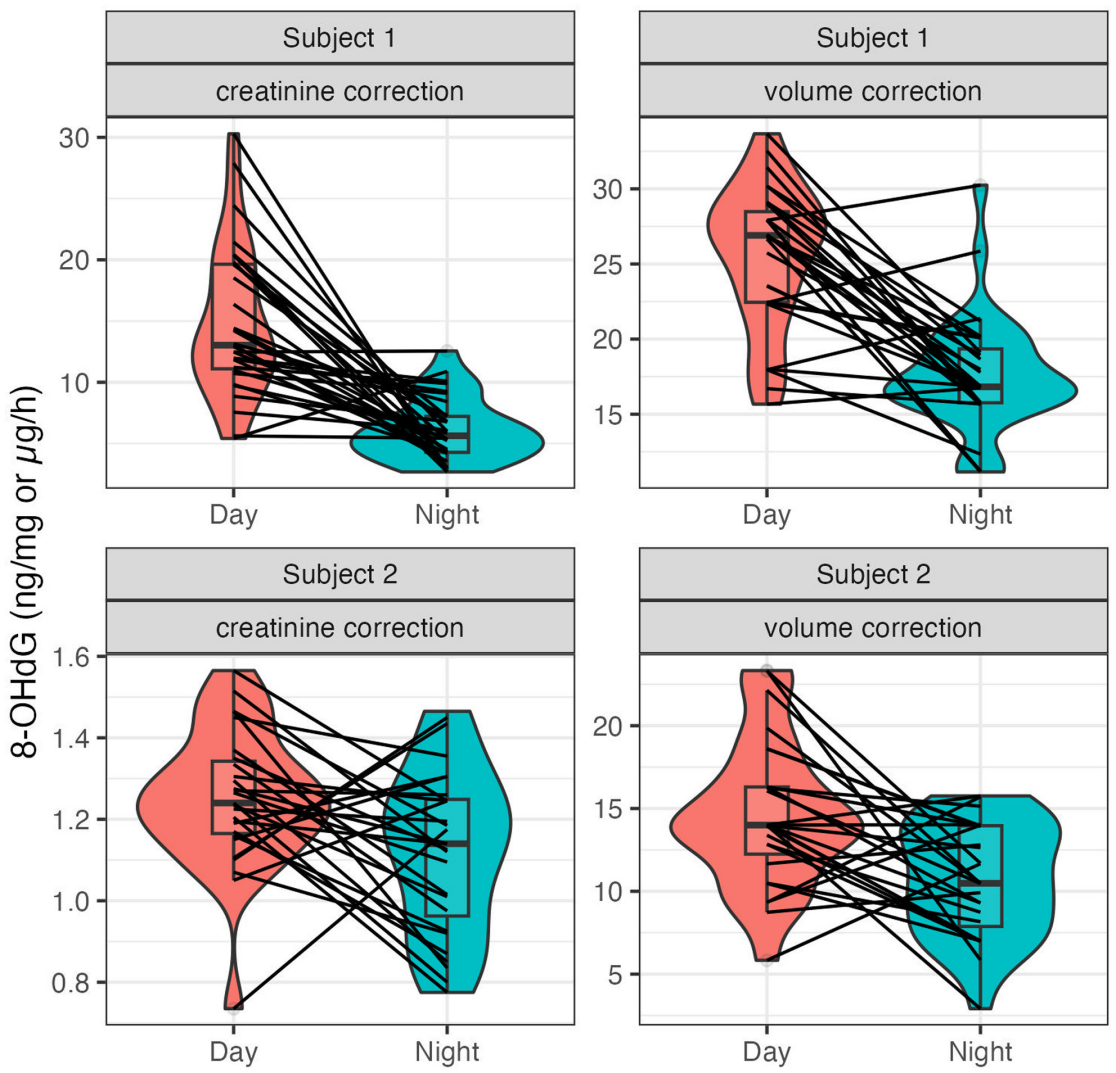
**Diurnal differences in urinary 8-OHdG**. Depicted is the difference between day and night values in urinary 8-OHdG as creatinine-corrected (left column) and volume-corrected (right column) values for subject 1 (top row) and subject 2 (bottom row). In each comparison, a statistically significant difference, as determined by paired, two-tailed t-tests, was found. Subject 1 n = 63, Subject 2 n = 55. Respective day-night pairs are connected. Boxplot displaying median with error bars representing the full range of data points without outliers. Density distribution displays the distribution of data points within the boxplot.

A significant difference was observed between day and night levels of urinary 8-OHdG with lower levels during the night for both subjects in creatinine-corrected (subject 1: t = –6.43, *p* < 0.01; subject 2: t = –2.69, *p* = 0.01) and volume-corrected values (subject 1: t = –7.30, *p* < 0.01; subject 2: t = –3.69, *p* < 0.01). Nevertheless, this day-night difference was not consistently expressed throughout the study period. During certain 24-h periods, an inverse relation occurred with higher values at night (Figure 3). Specifically, this was evident in 6.45% of creatinine-corrected and 12.9% of volume-corrected values for subject 1 and 22.22% of creatinine-corrected and 25.93% of volume-corrected values for subject 2. Furthermore, the significant day-night difference observed for 8-OHdG corrected by creatinine and urine volume was not found for the original (uncorrected) determinations of 8-OHdG and may originate from significant day-night differences characterizing both creatinine and urine volume.

The ACF plots of the uncorrected time series of 8-OHdG for subject 1 suggests the presence of an approximately 4-day periodic component, while a similar smaller peak is also seen for subject 2 (Supplementary Figure 1). ANOVA of 8-OHdG levels grouped in 4-day intervals confirms this pattern for subject 1 (*F* = 11.01, *p* < 0.01) and subject 2 (*F* = 2.78, *p* = 0.02). Statistically significant 4-day patterns can be observed for creatinine (*F* = 5.59, *p* < 0.01), urine volume (*F* = 8.84, *p* < 0.01), and 8-OHdG corrected by creatinine (*F* = 10.06, *p* < 0.01) or urine volume (*F* = 8.85, *p* < 0.01) in the case of subject 1, as well as for urine volume (*F* = 2.84, *p* = 0.02) and 8-OHdG corrected by urine volume (*F* = 2.83, *p* = 0.02) of subject 2. However, these ANOVA results for creatinine and urine volume reflect consistent day-night differences rather than a true 4-day cycle. This is supported by spectral analysis, which found no repeating pattern at the 4-day timescale for these parameters. In contrast, spectral analysis detects an approximately 4-day periodicity for the uncorrected urinary 8-OHdG levels. In the case of the uncorrected 8-OHdG data, a spectral peak is observed with a period between 90 to 102.9 h, accounting for approximately 50% and 21% of the total variance for subjects 1 and 2, respectively. At the common trial period of 90 h (3.75 days), phases for both subjects are similar (–282° and –276°, where 360° corresponds to 90 h, and 0° is the start of the time series). This component (*p* < 0.05 by cosinor) has a period slightly longer than a half a week. When analyzing daytime and nightime data separately, similar results are obtained (data not shown).

## Discussion

This study examined the day-night variations in urinary 8-OHdG concentrations in two breast cancer survivors over periods of 63 and 55 12-h intervals (i.e., 32 and 28 days), using 12-h pooled samples. The findings strongly indicate a day-night difference in corrected urinary 8-OHdG, with significantly higher levels during the day and lower levels during the night. This rhythm was consistent across both subjects and independent of the correction method used (creatinine or volume). Importantly, this study is the first to analyze continuous time series of this length, providing robust indications of significant day-night differences in corrected 8-OHdG series.

Inter-individual variations between subjects are observable in raw and corrected 8-OHdG levels, which can be attributed to various factors such as lifestyle, diet, exposure to environmental agents, and stress [8,19,37,38]. Reported values for urinary 8-OHdG levels vary across studies, with a recent meta-analysis reporting an average range of 5.9–21.6 ng 8-OHdG per mg creatinine for ELISA measurements in non-smoking subjects with a BMI <25 [38]. Notably, ELISA measurements generally overestimate 8-OHdG levels due to cross-reactivity (see Methods, [8,39]). Although the values observed in subject 2 fall below this reference, they align with findings from other studies [40]. Given that elevated 8-OHdG levels are commonly associated with breast cancer and tend to decrease during disease progression [1,8,19,41,42], levels within the commonly reported range might reflect a disease-free state in both subjects.

Despite the noted inter-individual differences, both subjects exhibit similar day-night variations in corrected 8-OHdG levels. The circadian rhythm of 8-OHdG has been investigated in prior studies, yielding inconsistent findings. Some studies reported circadian variation [43,44,45,46], while others did not observe such a rhythm [37,39,47,48,49,50], contrasting with our results. This discrepancy may stem from the comprehensive nature of our time series, comprising 63 and 55 continuous data points (i.e., 32 and 28 days) for the two subjects, and allowing for a more robust assessment of day-night differences. In contrast, many other studies focused on single 24-h [43,44,46,48,49,50] or 48-h measurements [47] or sampled only once per 24-h period [37]. Our data show that single days may not consistently follow the typical day-night pattern, with some periods showing higher 8-OHdG levels during the night (Figure 3). Thus, an extended measurement period is essential for accurately assessing the rhythm underlying urinary 8-OHdG levels. Additionally, forced nighttime sampling in some studies [43,44] may disrupt sleep patterns, potentially inducing psychological stress, which could influence 8-OHdG levels and dynamics [3,17,51,52]. Lastly, the observed daytime peaks and nighttime throughs of 8-OHdG align with previous studies in urine and saliva [45,46,48,49], though the use of 12-h urine samples limits the precise determination of peak and through timing beyond day-night differences.

When examining the day-night differences in 8-OHdG, it is important to take into account the subjects’ histories of breast cancer. Although both participants were considered disease-free at the start of this study [26,27], diseases have been shown to impact circadian rhythms [53,54]. Given the similar rhythmicity in 8-OHdG observed in both subjects, the disease’s impact on 8-OHdG rhythmicity may have been similar, negligible, or dissipated in their current healthy state. To better understand the impact of disease on 8-OHdG rhythmicity, conducting a similar analysis in individuals without a severe disease history would provide valuable insights. Notably, subject 2’s cancer-related amenorrhea likely did not exert a discernible effect on urinary 8-OHdG levels [51].

The difference in urinary 8-OHdG levels between day and night may be attributed to increased physical and metabolic activity during the day [55] or the influence of the circadian clock on biological functions. Lee et al. [56] observed elevated 8-OHdG levels during the nocturnal period in subjects exposed to illumination before sleep, suggesting a connection to the circadian clock. Importantly, 8-OHdG, as a marker of ‘whole-body’ oxidative damage, cannot provide specific insights into the cellular source of the ROS-induced damage [15]. Additionally, ROS can both cause DNA damage (leading to increased 8-OHdG excretion) and inhibit DNA repair (resulting in decreased 8-OHdG excretion) [15]. Without cellular insights, it is impossible to determine the cellular oxidative state solely based on urinary 8-OHdG levels. Manzella et al. [57] propose that the circadian rhythm of biomarkers of oxidative DNA damage may be influenced by the circadian activity of DNA repair systems, indicating an evolutionary adaptation to maximize protection against oxidative stress during the active phase of the day, resulting in higher excretion of 8-OHdG during that period.

Correcting raw measurements of urinary 8-OHdG is crucial to account for changes in urine concentration and prevent masking underlying dynamics [58]. As urinary samples exhibit significant variations based on hydration level [59], correction with an internal standard is essential [60], with creatinine levels or urinary volume per time commonly used as correction methods. However, both methods introduce some degree of uncertainty to the final result [61]. While creatinine correction can lead to higher uncertainty due to variations in creatinine levels independently of urinary flow, volume correction is more likely to introduce a bias towards lower values due to missed volumes [61]. As we show here, correction with either urine volume or creatinine excretion is necessary to reveal the observed day-night differences in 8-OHdG excretion, as this rhythm is not present in the uncorrected 8-OHdG time series for both subjects (Supplementary Figure 1). The uncorrected time series of 8-OHdG further shows an approximately 4-day periodicity, which falls within the half-week range. This component is similar to circasemiseptan components documented for other variables such as blood pressure, heart rate, melatonin, inflammatory parameters, or various cancer markers [62,63,64,65,66,67]. Further study is needed to understand the significance of this periodicity in uncorrected 8-OHdG time series. Taken together, this indicates that to correctly capture the circadian rhythmicity of urinary biomarkers, it is advisable to analyze both the corrected and uncorrected time series.

Recognizing the significance of day-night differences in urinary 8-OHdG is essential for valid sampling in clinical research and diagnostics. While first-morning void samples have been correlated with 24-h pooled urine [47], they do not capture circadian variations and thus, their validity remains uncertain [47,68]. This study was not designed to directly compare different sampling methods but aimed to investigate stress system dynamics under conditions of “life as it is lived.” The 12-h pooled urine samples were chosen over shorter interval sampling to reduce participant burden while maintaining high ecological validity, though they prevent analysis of finer timescale excretion dynamics. While the integrative single-case study design limits the participant numbers, resulting in a small sample size of n = 2, this approach offers a unique opportunity to track biochemical processes continuously and cumulatively in a setting of high ecological validity. This enables a more comprehensive dataset, revealing day-night differences in urinary 8-OHdG that designs averaging single measurements across individuals often miss.^1^ We argue that such reductionistic designs may overlook broader systemic effects and interactional patterns, leading to inconsistent or erroneous findings [28,29,30,31,62,66,69]. Future studies with larger cohorts can utilize mixed effects models to better understand inter-individual differences in the excretion patterns of 8-OHdG.

Notably, our coefficient of variation (CV) analysis revealed that longer sampling periods reduced variability, with higher CV for 12-h samples and decreasing as the sampling duration increased (Table 1). This suggests that longer sampling intervals may provide more reliable measurements by capturing the full spectrum of circadian fluctuations. Sampling methods that ignore these rhythmic variations may lead to false-positive or -negative results, potentially masking or exaggerating oxidative damage associated with diseases such as cancer, stroke, diabetes, and neurodegenerative disorders [19,70,71,72]. This, in turn, risks misdiagnosis, inappropriate treatment decisions, and missed opportunities for optimizing therapy timing based on circadian rhythms (chronotherapy) [73].

Understanding the diurnal variations of 8-OHdG in breast cancer survivors advances its potential use as a biomarker for breast cancer and enables a more precise assessment of oxidative stress changes during [74] and after cancer treatment. By tracking 8-OHdG as a marker of oxidative stress before, during, and after cancer treatment, clinicians could develop targeted interventions that account for circadian rhythms, potentially optimizing patient care and treatment strategies.

In conclusion, this investigation demonstrated circadian rhythms in the urinary 8-OHdG time series of two former breast cancer patients using the integrative-single case design tailored to the biopsychosocial characteristics of “life as it is lived” conditions [28,29,30]. Further studies in patients and healthy volunteers must now follow in order to strengthen these findings and to investigate the mechanisms linking circadian rhythms, oxidative stress, and disease pathology. This could ultimately contribute to a comprehensive understanding of 8-OHdG dynamics, informing both diagnostic approaches and therapeutic strategies for ROS-damage-related pathologies such as cancer.

## Additional Files

The additional files for this article can be found as follows:

10.5334/jcr.252.s1 Supplemental Figure 1. Scoping Review Search.

10.5334/jcr.252.s2 Supplemental Material. Supplementary raw data.

## Footnotes

By storing multiple aliquots of each 12-h interval’s urine, we created a biobank that we are eager to share with the research community for further analysis and hypothesis-testing of biomarker dynamics in breast cancer research.

## Abbreviations

ROS – Radical Oxygen Species

8-OHdG – 8-hydroxy-2’-deoxyguanosine

ELISA – Enzyme-linked Immunosorbent Assay

CV – Coefficient of variation

uCr – Urinary creatinine

ACF – Auto Correlation Function
