# Supplementary material for: Diurnal Variation of 8-hydroxy-2’-deoxyguanosine in Continuous Time Series of Two Breast Cancer Survivors
Source: J Circadian Rhythms. 2025 May 19;23:6. doi: 10.5334/jcr.252 (PMC12101107; doi:10.5334/jcr.252)
Supplement: Supplemental Figure 1. — Scoping Review Search. [file jcr-23-252-s1.pdf]

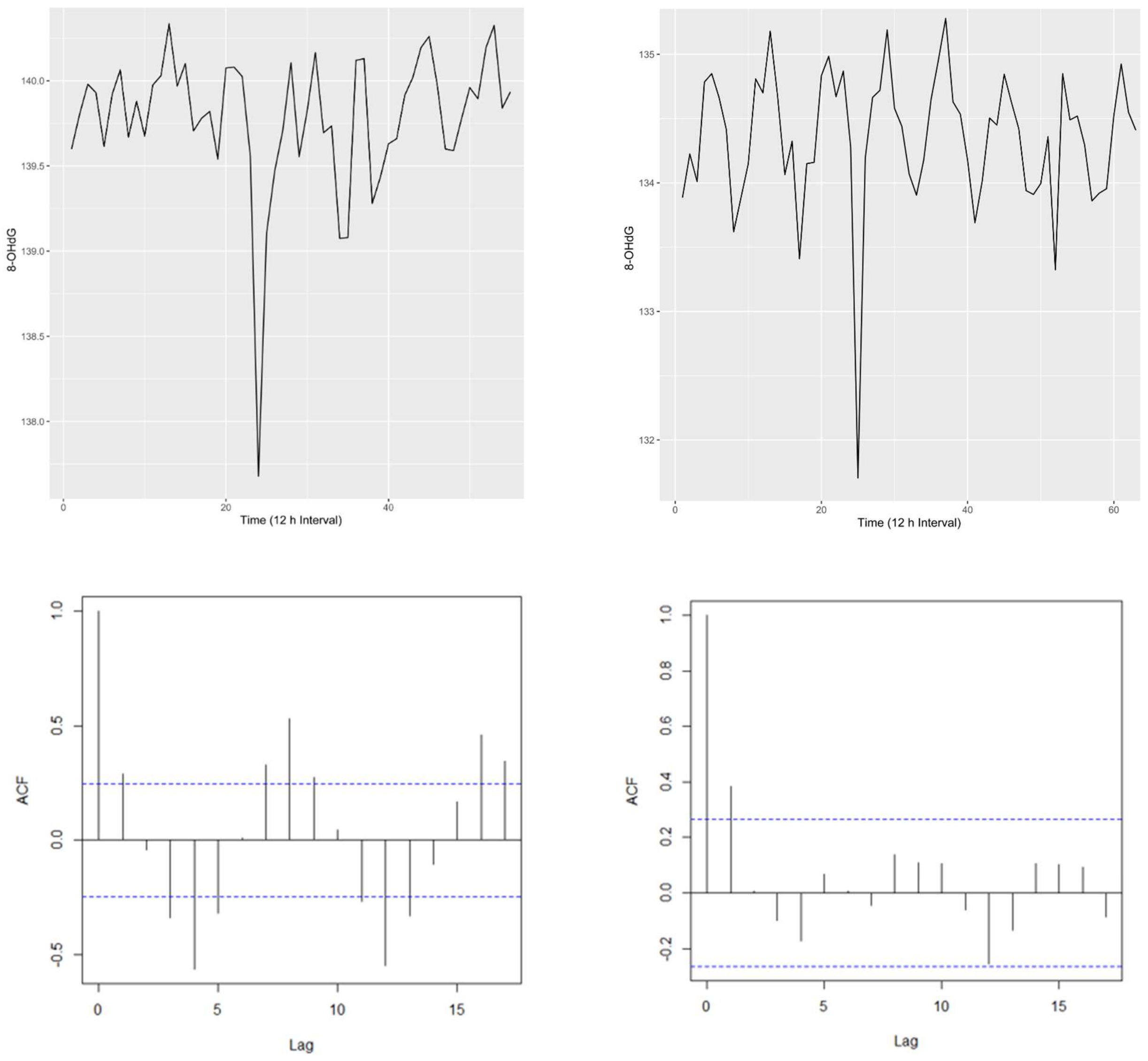

Supplementary Figure 1. Uncorrected time series of 8-OHdG (top row) for subjects 1 (left) and 2 (right) with corresponding ACF plots (bottom row).
